# Assessing the effectiveness of Chagas disease education for healthcare providers in the United States

**DOI:** 10.1186/s12879-020-05474-w

**Published:** 2020-10-09

**Authors:** Paula Stigler Granados, Gerardo J. Pacheco, Evangelina Núñez Patlán, Jose Betancourt, Lawrence Fulton

**Affiliations:** School of Health Administration, Texas State University, 601 University Dr, San Marcos, TX 78666-4606 USA

**Keywords:** Chagas disease, Neglected tropical diseases, *Trypanosoma cruzi*, Online medical education, Telehealth

## Abstract

**Background:**

Chagas disease is a zoonotic infection caused by the parasite *Trypanosoma cruzi,* which affects an estimated 8–11 million people globally. **Chagas disease** is almost always associated with poverty in rural areas and disproportionately impacts immigrants from Latin America living in the United States. Approximately 20–30% of people who are infected with Chagas disease will develop a chronic form of the infection that can be fatal if left untreated. Chagas disease is vastly underestimated in the United States, often goes undiagnosed and is not well understood by most U.S. healthcare providers. One of the most important ways at reducing barriers to improving diagnostics of Chagas disease in the U.S. is giving healthcare providers the most up-to-date information and access to leading experts.

**Methods:**

An online webinar was conducted for healthcare providers, veterinarians and public health professionals using Chagas disease expert panelists. Pre and post tests were administered to participants (*n* = 57) to determine the efficacy in raising awareness and to determine key focus areas for improving knowledge. A Wilcoxon rank-sum was used for non-parametric variables equivalent and for questions that assessed knowledge the McNemar’s Chi-Square test was used.

**Results:**

There were statistically significant learning increases in multiple categories including transmission (p = <.001), clinical presentation (*p* = 0.016), diagnostics (p = <.001), and treatment (p = <.001).

**Conclusion:**

Providing easily accessible learning opportunities using validated testing and evaluations should be further developed for rural healthcare providers in the U.S. as well as healthcare providers serving under represented populations such as immigrants. There is a clear lack of knowledge and awareness surrounding Chagas disease in the United States and just by raising awareness and providing education on the topic, lives will be saved.

## Background

Chagas disease is a zoonotic infection caused by the parasite *Trypanosoma cruzi (T.cruzi),* which affects an estimated 8–11 million people globally [1–8]. Approximately 20–30% of people who are infected with Chagas disease will develop a chronic form of the infection that can lead to fatal heart or gastrointestinal diseases [5, 9, 10]. Although Chagas disease is most commonly found in Latin American countries, there has been a growing concern that it is vastly underestimated in the United States [1–5, 9, 11] Estimates that use modeled data suggest that there are between 326,000 and 347,000 individuals born in Latin America living in the United States that are infected with *T.cruzi* [2, 3, 12]. This population may hold the highest burden of Chagas disease in the United States due to a higher prevalence in their countries of origin [13, 14]. However, in the southern United States there have been an increasing number of autochthonous and congenital transmission cases being reported since 2000 [3, 15–19]. It is generally believed that transmission rates in the United States are lower than in Latin America, mainly because of housing conditions and different vector behaviors [16, 20]. Nevertheless, there are 29 out of 50 states reporting triatomine insects with 10 of the 11 species found to have the ability to be infected with the parasite [15, 21, 22]. In several studies of triatomines collected in Texas, more than 60% were infected with *T. cruzi* and of those infected many had human blood in their gut [22–26]. Many of these studies suggest that locally acquired infections in the United States may occur more frequently than previously thought, however due to a lack of knowledge about Chagas disease in the United States it is often overlooked [18, 27].

Regardless of where a person is infected, less than 1% of people in the United States will ever receive a clinical diagnosis of Chagas disease and even fewer will ever receive treatment [4, 12]. This lack of diagnosis and treatment is a result of a combination of factors including an absence of awareness by healthcare providers, limited diagnostic tools, and access to healthcare by the populations most vulnerable to this disease [12, 28, 29]. According to Lynn, et al., they found that shared risk factors for transmission of Chagas disease in the United States were living in a rural area and/or a history of outdoor activities or work [15]. This leads us to conclude that healthcare providers serving populations in rural areas or underserved migrant population are a good starting point to target Chagas disease education efforts [12, 29–31].

As of 2017, only six states participate in surveillance and reporting of Chagas disease [12, 31, 32]. Educational materials on Chagas disease may be found online by several different sources and from the individual state health departments and the Centers for Disease Control and Prevention (CDC). Despite the availability of educational materials, a study on patient perspectives and access to Chagas disease in the United States emphasizes low awareness among providers and nonexistent health education campaigns as barriers to treatment [29]. In 2015, the CDC funded several projects to raise awareness and improve knowledge of Chagas disease among healthcare providers in the United States. As a result, the Texas Chagas Taskforce (TCTF) was developed and focused on using a One Health approach to information dissemination [25, 33]. The TCTF recognized the need for a structured education program and therefore developed an online webinar on Chagas disease targeting healthcare providers, veterinarians and public health professionals. The purpose of this paper is to analyze the efficacy of an online education program for health professionals in raising awareness of Chagas disease and to determine key focus areas for improving knowledge on this complex disease.

## Methods

A voluntary group of individuals from healthcare and veterinary medicine backgrounds participated in a 2-h online webinar about Chagas disease. Of the participants, *n* = 57 completed both the pre and post-test knowledge assessment (see Appendix 1 and 2). The session was conducted online during the afternoon and consisted of six individual presentations by Chagas disease experts, including: physicians, veterinarians and epidemiologists at the state and national levels. The presentations were followed by time for questions and answers from participants. The targeted subjects for study recruitment were healthcare providers, veterinarians and public health professionals, most of whom were practicing in Texas. A promotional flyer was distributed via emails and social media and university list serves. An online event registration site was used to register participants and an informed consent notice was included in the pre-knowledge test, which participants were asked to agree or disagree with before proceeding with the study. This study was reviewed and approved by the UTHealth Institutional Review Board.

Qualtrics software [34] was chosen as the survey tool to host the pre- and post-knowledge tests as well as the evaluation of training. WebEx software was utilized as the host teleconferencing platform. After registration, participants received an email with the Qualtrics link to a pre-knowledge test and the link to the online webinar. Email reminders with the link were sent to the participants one week and again one day prior to the training session. The pre-knowledge test consisted of 16 questions (12 knowledge and 4 demographic) and was to be completed prior to the session. The post-knowledge test was the same as the pre-knowledge test with the same 12 knowledge questions. Both the pre and post knowledge questions were developed and piloted with Chagas disease experts from the TCTF and based on information provided in an online course from the Centers for Disease Control and Prevention [35]. An evaluation of the training survey given upon completion of the webinar was given and consisted of 20 questions addressing occupation, program experience, and program content. Both the post-knowledge test and the evaluation were to be completed after the last session. A total of 239 participants registered for the sessions; however, only 146 participated in the webinar and of those only 57 completed both the pre and post tests. Continuing education credit was given to those participants that attended and completed both tests. There were 159 continuing education credits provided (51 CMEs and 104 CEUs).

Two different statistical tests evaluated the efficacy of the educational event. For self-reported knowledge gains (e.g., how would you describe your knowledge of Chagas disease and how confident are you that your knowledge is up to date), Wilcoxon rank-sum tests were used. A Wilcoxon rank-sum was used as a non-parametric equivalent to the paired t-test, which was appropriate as the data were not quantitative. For questions that assessed actual knowledge, McNemar’s Chi-Square test was used. This test was deemed appropriate to evaluate correct / incorrect responses before and after session. The analytical software for the study was R Statistical Software [36] and Microsoft.

## Results

Of the146 attendees, 25% were doctors of veterinary medicine, 12% medical doctors or doctors of osteopathy, 10% nurses, and 54% were other healthcare or public health professionals. Of those completing both the pre- and post-tests, 25% were doctors of veterinary medicine, 14.3% were medical doctors or doctors of osteopathy, 8.9% were nurses, and the remaining 51.8% were other healthcare or public health professionals. Table 1 provides the means and standard deviations for each of the questions (truncated item) on the knowledge assessment. The table provides 1) the results for the pre- and post-tests, 2) the difference between the two tests, and 3) a 95% confidence interval for the difference.

**Table 1:** Chagas disease (CD) knowledge pretest, posttest with difference means / standard deviations by survey question

	Pre	Post	Pre-Post	Pre	Post	Pre-Post	Lower 95%	Upper 95%
Item (*n* = 56)	Mean	Mean	Mean	SD	SD	SD	Post-Pre	Post-Pre
Describe your level of CD Knowledge 1 (I don’t know anything); 2 (Very limited); 3 (Limited); 4 (Good); 5 (Excellent)	3.13	3.95	0.82	0.95	0.82	0.79	0.61	1.03
How confident are you with your CD Knowledge is current? 1 (Don’t know); 2 (Not at all confident); 3 (Somewhat confident); 4 (Confident); 5 (Very confident)	2.84	3.96	1.13	1.01	0.93	1.08	0.84	1.41
CD is present in Texas? (True or False)	0.96	0.96	0.00	0.19	0.19	0.30	−0.08	0.08
Cause of CD	0.84	0.91	0.07	0.37	0.29	0.26	0.00	0.14
Transmission of CD	0.30	0.59	0.29	0.46	0.50	0.49	0.16	0.42
What part of the world is CD transmitted?	0.73	0.91	0.18	0.45	0.29	0.39	0.08	0.28
What % of patients develop clinical disease?	0.39	0.70	0.30	0.49	0.46	0.54	0.16	0.44
CD Symptoms	0.80	0.88	0.07	0.40	0.33	0.37	−0.03	0.17
CD Clinical Manifestations	0.73	0.89	0.16	0.45	0.31	0.42	0.05	0.27
Methods to diagnose CD	0.41	0.86	0.45	0.50	0.35	0.54	0.31	0.59
CD Treatment	0.52	0.77	0.25	0.50	0.43	0.44	0.14	0.36
EKG Typical of CD	0.38	0.48	0.11	0.49	0.50	0.56	−0.04	0.25

Starting at item 3 in Table 1 (“Present in Texas?”), the values are associated with correct or incorrect answers and may be interpreted as percentages. Prior to the training, 96% were correct in understanding that the disease was present in Texas, and 84% knew that the disease was caused by a parasite rather than a virus, bacterium, or other. Only 30% knew that *T. cruzi*, the parasite that causes Chagas disease was not transmitted by the saliva of an infected triatome (but rather the feces). While 73% knew that Chagas disease was prevalent in the Southern United States, only 39% could identify that 21–40% of those infected develop clinical disease. Eighty percent knew the symptoms prior to training, and 73% identified the clinical manifestations with only 41% correctly identifying the laboratory methods used to diagnose Chagas disease. Fifty-two percent knew the treatment recommendations, and only 38% understood EKG findings associated with Chagas disease.

**Fig. 1:**
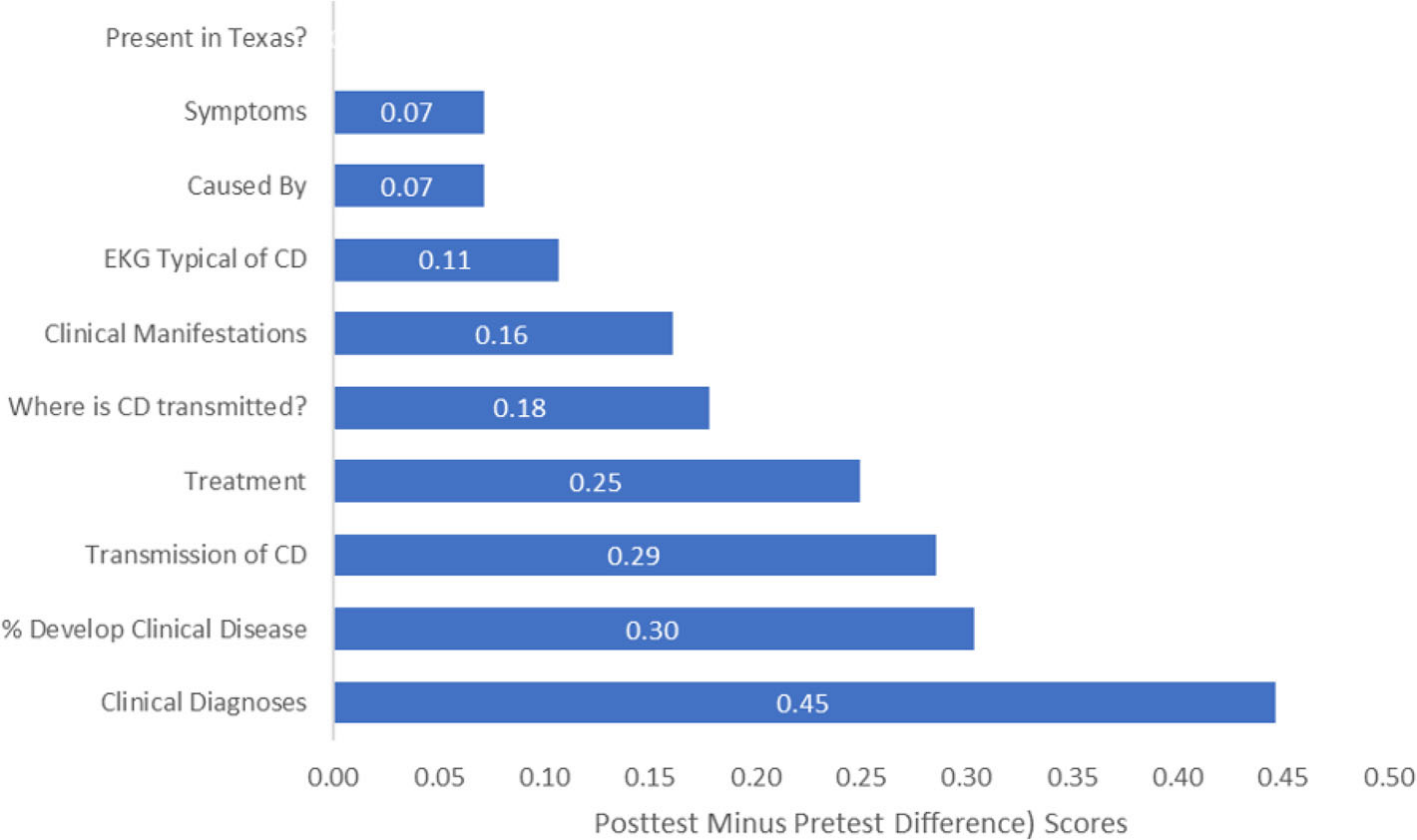
Graph of the Chagas disease knowledge post-test less the pre-test scores

Figure 1 graphs the post-test less the pre-test mean scores. From this figure, it is clear that there were knowledge improvements for all questions except for one: True or False: Chagas disease is present in Texas.

A Wilcoxon rank sum test of self-assessed Chagas disease knowledge (“How would you describe your level of knowledge about Chagas disease?”) indicated a significant difference in pre-test versus post-test (V = 35, *p* < .001). Self-assessed Chagas disease knowledge improved from a median of 3 to a median of 4. Another Wilcoxon rank sum test evaluated “How confident are you that your knowledge on Chagas disease is up to date?” Again, the results were statistically significant (V = 39, *p* < .001).

McNemar Chi-Square tests were run for each of the knowledge questions in the survey. Table 2 provides the results. Pre-post statistically significant differences were found in 6 of the 10 questions including questions about how Chagas disease is transmitted (*p* < .001), where it is transmitted via vectors (*p* < .005) what percentage of infected individuals develop clinical disease (p < .001), what clinical manifestations occur (*p* = .016), laboratory methods of how Chagas disease is diagnosed (p < .001), and how Chagas disease is treated (p < .001). Of the 56 attendees, 96% knew in advance that Chagas disease was present in Texas, and that remained static after training. There were increases in knowledge for all other survey questions, even for those questions that were not statistically significant. Table 1 shows the post-pre-test mean differences.

**Table 2:** Chagas disease pre-post knowledge analysis of questions in survey

Question	McNemar c^2^_1_	*p*-value
Present in Texas?	0.000	1.000
Cause of CD	2.250	0.134
Transmission of CD	12.500	<.001*
Where is CD transmitted?	8.100	<.005*
% Develop Clinical Disease	12.190	<.001*
Symptoms	1.125	0.289
Clinical Manifestations	5.818	0.016*
Methods to diagnose CD	21.333	<.001*
Treatment	12.071	<.001*
EKG Typical of CD	1.388	0.239

*Statistically significant at a value of <.05

## Discussion

The value and acceptability of webinar platforms to deliver training to groups is well-documented, often with good learning outcomes [37–43]. As the technology and tools that facilitate delivery of training through this evolving medium continue to mature, those seeking to raise awareness of emerging health threats such as Chagas disease recognize the invaluable worth of embracing this technology and assessment of learning [44]. The results of the pre- and post-test evaluations in this study indicate an increase in overall knowledge of Chagas disease among the healthcare professionals who participated in the webinar. Prior research highlights the relatively low number of studies that evaluate the awareness of Chagas disease among healthcare professionals, particularly those working with migrants from Latin America or who are physically located in Chagas-endemic areas [44]. These studies are critical in assessing the knowledge, skills, and abilities of healthcare providers to recognize and identify this hidden but growing threat, correctly diagnose it and offer proper treatment. As with any disease or health condition that burdens a population, the crucial first step in addressing it is correctly *recognizing* and *identifying* its presence. The results of the pre-test indicate that while most of those who took the pre-test understood that Chagas disease is found in Texas, the extent of their knowledge of Chagas disease beyond this fact was minimal, particularly the poor, initial score on the ‘clinical diagnosis’ question. This is particularly concerning for those providers who encounter a patient with potential signs and symptoms of exposure to the bite of an infected triatomine insect or have a high-risk patient and may elect to dismiss the real possibility of a Chagas disease diagnosis.

Raising the awareness of healthcare providers and professional in other areas such as veterinary medicine and public health of the presence of Chagas disease in what has historically been considered non-endemic regions will aid them in asking the right questions of their patients/clients/populations. Additionally, the pre-test answers to the questions ‘transmission of Chagas disease’ and ‘percent development of Chagas disease’ indicate an insufficient preparedness of the provider to inform and educate their patient on what to look for in their homes or work places to see if they may have elevated risks of exposure to the vector.

Limitation(s): Although a total of 240 participants registered for and attended the sessions, the actual number of participants who completed both the pre and post-test knowledge assessment was 57. This relatively small sample size may not be representative of the population and study findings may not be representative of the population. Additionally, the study may be subject to self-selection bias as those who responded to the offer of participating in the training, and who successfully completed it may illustrate a higher level of interest in Chagas disease over the population of providers in general. Unlike other infectious diseases, no validated tool existed at the time of this webinar to assess healthcare providers’ knowledge on Chagas disease. Therefore, the questionnaire used was not a comprehensive tool and mainly focused on providers in Texas.

## Conclusion

These findings illustrate the need to educate healthcare providers and other public health professionals on Chagas disease, as it is a complex disease that is not well understood in the United States. Increasing awareness, especially among those providers in rural areas and serving populations such as recent immigrants is a high priority. However, face-to-face educational opportunities for this group of professionals may not be possible or could be cost-prohibitive. The webinar demonstrated significant knowledge increases in several areas and proved to be effective in disseminating information from experts on the subject. We recommend that future planning for continued educational online platforms and programs be continued and improve the target audiences. Incorporating public health / community health workers into these trainings could also help make an impact on ‘arming’ populations at risk with the knowledge and tools to better protect themselves against this silent threat.

## Supplementary information

**Additional file 1.**

**Additional file 2.**

## Data Availability

The datasets used and/or analyzed during the current study are available from the corresponding author on reasonable request.

## Abbreviations

*T.cruzi*: *Trypanosoma cruzi*
CDC: Centers for Disease Control and Prevention
TCTF: Texas Chagas Taskforce
CD: Chagas disease
