# Supplementary material for: Assessing the effectiveness of Chagas disease education for healthcare providers in the United States
Source: BMC Infect Dis. 2020 Oct 9;20:743. doi: 10.1186/s12879-020-05474-w (PMC7547496; doi:10.1186/s12879-020-05474-w)
Supplement: Supplementary file 1 — Additional file 1. [file 12879_2020_5474_MOESM1_ESM.pdf]

CME Chagas Disease Workshop: Pre-Session Questionnaire

1. Please indicate the profession that best describes you:
   MD/ DO
   DVM
   Other: ____

If you are **not** a practicing physician (i.e., MD/ DO) please skip to Question 5

2. What is your primary specialization?
   Infectious disease
   Obstetrics/ gynecology
   Cardiology
   General practitioner/ family physician
   Other: ____

3. What is your primary type of medical practice?
   Private
   Teaching hospital
   Community hospital
   Other: ____

4. How many years have you been a practicing physician?
   Less than 3 years
   3 to 5 years
   5 to 10 years
   More than 10 years

5. How would you describe your level of knowledge about Chagas disease?
   Excellent
   Good
   Limited
   Very limited
   I don't know anything about Chagas Disease

6. How confident are you that your knowledge on Chagas disease is up to date?
   Very confident
   Confident
   Somewhat confident
   Not at all confident

7. True or False: Chagas disease is present in Texas.
   True
   False

8. Chagas disease is caused by a (select one answer):
   Bacteria
   Virus
   Parasite
   Fungus
   I don't know

ID:________________

9. True or False: The parasite that causes Chagas disease, *T cruzi*, is transmitted by the saliva of an infected triatomine bugs when they bite a person, typically at night
   - True
   - False

10. In what parts of the world is Chagas disease transmitted by vector bugs (choose the best answer)?
   - East Asia and parts of Africa
   - Cuba and Puerto Rico
   - Southern United States, Mexico, Central America, and South America
   - Central America, Mexico, and parts of Africa

11. Approximately, what percentage of patients with chronic Chagas infection eventually develop clinical disease?
   - <20%
   - 21-40%
   - >40%
   - Don’t know

12. Chagas disease symptoms are?
   - Acute for several weeks then immediately symptomatic
   - Acute for several week; asymptomatic for years to decades then sometimes symptomatic
   - There are no symptoms
   - Don’t know

13. People with chronic Chagas disease may have (circle all that apply)?
   - Cardiac conduction abnormalities
   - Cardiomyopathy
   - Megacolon
   - Co-clinical manifestations
   - Don’t know

14. What methods may be used to diagnose Chagas disease?
   - Blood smear
   - PCR assays
   - Serologic tests
   - First and Third choices
   - All of the above

15. Should patients with chronic Chagas disease be treated with antitrypanosomal drugs?
   - No, there is no evidence that antitrypanosomal treatment for chronic Chagas disease can be effective
   - Only patients less than 5 years old should be treated for chronic Chagas disease.
   - Treatment is always recommended for patients up to age 18 years and generally recommended for patients aged 18-50
   - Only Chagas disease patients older than 50 years should be offered antitrypanosomal treatment.

16. What are EKG findings typical of Chagas cardiomyopathy?
   - Right bundle branch block
   - Left anterior fascicular block
   - First degree AV block
   - The First and Third answers
   - All of the above

ID:_________________
