# Supplementary material for: Assessing the effectiveness of Chagas disease education for healthcare providers in the United States
Source: BMC Infect Dis. 2020 Oct 9;20:743. doi: 10.1186/s12879-020-05474-w (PMC7547496; doi:10.1186/s12879-020-05474-w)
Supplement: Supplementary file 2 — Additional file 2. [file 12879_2020_5474_MOESM2_ESM.pdf]

CME Chagas Disease Workshop: Post-Session Questionnaire

1. How would you describe your level of knowledge about Chagas disease?
   Excellent
   Good
   Limited
   Very limited
   I don't know anything about Chagas Disease

2. How confident are you that your knowledge on Chagas disease is up to date?
   Very confident
   Confident
   Somewhat confident
   Not at all confident

3. True or False: Chagas disease is present in Texas.
   True
   False

4. Chagas disease is caused by a (select one answer):
   Bacteria
   Virus
   Parasite
   Fungus
   I don't know

5. True or False: The parasite that causes Chagas disease, *T cruzi*, is transmitted by the saliva of an infected triatomine bugs when they bite a person, typically at night
   True
   False

6. In what parts of the world is Chagas disease transmitted by vector bugs (choose the best answer)?
   East Asia and parts of Africa
   Cuba and Puerto Rico
   Southern United States, Mexico, Central America, and South America
   Central America, Mexico, and parts of Africa

7. Approximately, what percentage of patients with chronic Chagas infection eventually develop clinical disease?
   <20%
   21-40%
   >40%
   Don't know

8. Chagas disease symptoms are?
   Acute for several weeks then immediately symptomatic
   Acute for several week; asymptomatic for years to decades then sometimes symptomatic
   There are no symptoms
   Don't know

9. People with chronic Chagas disease may have (circle all that apply)?

ID:________________

Cardiac conduction abnormalities
Cardiomyopathy
Megacolon
Co-clinical manifestations
Don't know

10. What methods may be used to diagnose Chagas disease?
    - Blood smear
    - PCR assays
    - Serologic tests
    - First and Third choices
    - All of the above

11. Should patients with chronic Chagas disease be treated with antitrypanosomal drugs?
    - No, there is no evidence that antitrypanosomal treatment for chronic Chagas disease can be effective
    - Only patients less than 5 years old should be treated for chronic Chagas disease.
    - Treatment is always recommended for patients up to age 18 years and generally recommended for patients aged 18-50
    - Only Chagas disease patients older than 50 years should be offered antitrypanosomal treatment.

12. What are EKG findings typical of Chagas cardiomyopathy?
    - Right bundle branch block
    - Left anterior fascicular block
    - First degree AV block
    - The First and Third answers
    - All of the above

ID:_______________
